# Complete Genome Sequences of Two Hydrangea Ringspot Virus Isolates from Japan

**DOI:** 10.1128/genomeA.00022-16

**Published:** 2016-03-31

**Authors:** Akira Yusa, Nozomu Iwabuchi, Hiroaki Koinuma, Takuya Keima, Yutaro Neriya, Masayoshi Hashimoto, Kensaku Maejima, Yasuyuki Yamaji, Shigetou Namba

**Affiliations:** Laboratory of Plant Pathology, Department of Agricultural and Environmental Biology, Graduate School of Agricultural and Life Sciences, The University of Tokyo, Tokyo, Japan

## Abstract

Hydrangea ringspot virus (HdRSV) is a plant RNA virus, naturally infecting *Hydrangea macrophylla*. Here, we report the first genomic sequences of two HdRSV isolates from hydrangea plants in Japan. The overall nucleotide sequences of these Japanese isolates were 96.0 to 96.3% identical to those of known European isolates.

## GENOME ANNOUNCEMENT

Members of the genus *Potexvirus* in the family *Alphaflexiviridae* are nonenveloped, filamentous, flexuous plant viruses (470 to 580 × 13 nm), with a monopartite, single-stranded positive-sense RNA genome. They occur worldwide and are thought to be transmitted in nature, mainly by mechanical contact (1). Hydrangea ringspot virus (HdRSV) belongs to the genus *Potexvirus*, and naturally infects *Hydrangea macrophylla*, a deciduous shrub native to Japan. To date, complete genome sequences of two HdRSV isolates (PD 109 [AY707100] and HydCZ [GQ265901]) from European countries have been determined (2, 3). In Japan, although HdRSV infection was once reported from imported hydrangea plants (4), no sequence information of Japanese HdRSV isolates has been available prior to this report.

In 2011, we found one hydrangea plant with yellow ringspots on systemic leaves, and another with chlorotic ringspots on lower leaves in Japan. From both plants, potexvirus-like virus particles were observed by electron microscopy, suggesting that they might be infected by HdRSV. Total RNA was extracted using ISOGEN (Nippon Gene, Japan) and served as templates for reverse transcription-PCR (RT-PCR) with primer pairs Hd24F (5′-GAAAAGTTCCACACCCAAACCAAA-3′)/Hd3612R (5′-CTGGGATTGGTCGAAGGCGGTGAA-3′) and Hd3109F (5′-AGCCCTCCTCTGGGAGACAATCAA-3′)/oligo-dT, designed on the basis of genome sequences of PD 109 and HydCZ. The two resultant overlapping RT-PCR fragments were sequenced and assembled. The 5′ terminal sequences were determined using the 5′ RACE system for rapid amplification of cDNA ends, version 2.0 (Invitrogen, USA). We eventually obtained two complete genome sequences of HdRSV, and named these isolates Gu1 and Gu2.

The complete genomes of isolates Gu1 and Gu2 consisted of 6,194 and 6,196 nucleotides (nt), respectively, excluding the poly (A) tail, with a 2-nt difference in the 3′ untranslated region. They contained six open reading frames, putatively encoding RNA-dependent RNA polymerase (RdRp; nt 96 to 4,259), triple gene block protein 1 (TGBp1; nt 4,262 to 4,963), TGBp2 (nt 4,920 to 5,264), TGBp3 (nt 5,149 to 5,370), coat protein (CP; nt 5,395 to 6,072), and an uncharacterized protein (nt 5,555 to 6,028). Pairwise analysis revealed that Gu1 and Gu2 shared 98.3 and 99.1% nucleotide identity for RdRp and CP sequences, respectively. In addition, the nucleotide sequences of RdRp and CP of Gu1 were 95.4 to 95.5% and 96.4 to 96.5% identical, respectively, to those of the European isolates of HdRSV. According to the species demarcation criteria in the *Alphaflexiviridae* (1), Gu1 and Gu2 were identified as isolates of HdRSV.

Alignment of the HdRSV genomes indicated that three nucleotide insertions, at positions 1,092, 1,107, and 1,162 in Gu1 and Gu2, caused a 23 amino acid (aa) frameshift in the methyltransferase–helicase interdomain region of RdRp. Similar nucleotide insertions were also observed at positions 4,510, 4,582, and 4,583 in Gu1 and Gu2, producing a 25 aa frameshift overlapping a helicase motif of TGBp1. These frameshifted aa regions were highly conserved among the Japanese isolates of HdRSV and other potexviruses, but not among the Japanese and European isolates of HdRSV.

### Nucleotide sequence accession numbers.

The viral genome sequences were deposited in GenBank under the accession numbers LC107516 (Gu1) and LC107517 (Gu2).
